# *In silico* approaches for drug repurposing in oncology: a scoping review

**DOI:** 10.3389/fphar.2024.1400029

**Published:** 2024-06-11

**Authors:** Bruno Raphael Ribeiro Cavalcante, Raíza Dias Freitas, Leonardo de Oliveira Siquara da Rocha, Roberto de Souza Batista Dos Santos, Bruno Solano de Freitas Souza, Pablo Ivan Pereira Ramos, Gisele Vieira Rocha, Clarissa Araújo Gurgel Rocha

**Affiliations:** ^1^ Gonçalo Moniz Institute, Oswaldo Cruz Foundation (IGM-FIOCRUZ/BA), Salvador, Brazil; ^2^ Department of Pathology and Forensic Medicine of the School of Medicine, Federal University of Bahia, Salvador, Brazil; ^3^ Department of Social and Pediatric Dentistry of the School of Dentistry, Federal University of Bahia, Salvador, Brazil; ^4^ D’Or Institute for Research and Education (IDOR), Salvador, Brazil; ^5^ Center of Data and Knowledge Integration for Health (CIDACS), Salvador, Brazil; ^6^ Department of Propaedeutics, School of Dentistry of the Federal University of Bahia, Salvador, Brazil

**Keywords:** drug repurposing, drug repositioning, cancer, *in silico* methods, scoping review

## Abstract

**Introduction:** Cancer refers to a group of diseases characterized by the uncontrolled growth and spread of abnormal cells in the body. Due to its complexity, it has been hard to find an ideal medicine to treat all cancer types, although there is an urgent need for it. However, the cost of developing a new drug is high and time-consuming. In this sense, drug repurposing (DR) can hasten drug discovery by giving existing drugs new disease indications. Many computational methods have been applied to achieve DR, but just a few have succeeded. Therefore, this review aims to show *in silico* DR approaches and the gap between these strategies and their ultimate application in oncology.

**Methods:** The scoping review was conducted according to the Arksey and O’Malley framework and the Joanna Briggs Institute recommendations. Relevant studies were identified through electronic searching of PubMed/MEDLINE, Embase, Scopus, and Web of Science databases, as well as the grey literature. We included peer-reviewed research articles involving *in silico* strategies applied to drug repurposing in oncology, published between 1 January 2003, and 31 December 2021.

**Results:** We identified 238 studies for inclusion in the review. Most studies revealed that the United States, India, China, South Korea, and Italy are top publishers. Regarding cancer types, breast cancer, lymphomas and leukemias, lung, colorectal, and prostate cancer are the top investigated. Additionally, most studies solely used computational methods, and just a few assessed more complex scientific models. Lastly, molecular modeling, which includes molecular docking and molecular dynamics simulations, was the most frequently used method, followed by signature-, Machine Learning-, and network-based strategies.

**Discussion:** DR is a trending opportunity but still demands extensive testing to ensure its safety and efficacy for the new indications. Finally, implementing DR can be challenging due to various factors, including lack of quality data, patient populations, cost, intellectual property issues, market considerations, and regulatory requirements. Despite all the hurdles, DR remains an exciting strategy for identifying new treatments for numerous diseases, including cancer types, and giving patients faster access to new medications.

## 1 Introduction

Cancer is a term that refers to a group of diseases that occur when cells divide uncoordinatedly with other tissues and do not respond appropriately to the signals that control cellular behavior (Sarkar et al., 2013). This feature happens as a result of multifactorial molecular events that involve interactions between the genes and the environment of an organism through a process called carcinogenesis (Karidio and Sanlier, 2021). As the disease progresses, those aberrant cells may invade and ultimately colonize other tissues and neighboring normal organs in a process called metastasis, which is a cancer hallmark that accounts for the most significant number of cancer-related deaths (Fares et al., 2020).

Given the diverse complexity of cancer phenotypes and genotypes, researchers have faced significant challenges in identifying optimal chemical entities as therapeutic agents for each type of cancer, aiming to halt disease progression at both molecular and physiological levels. However, the scarcity of approved drugs remains burdensome and requires discovering new therapeutic molecules.

Developing a new drug is expensive and time-consuming. Indeed, bringing a single medicine from scratch to pharmacy shelves ranges from US$ 944 million to US$ 2.8 billion (adjusted to 2019 prices) (Simoens and Huys, 2021) and takes approximately 10–15 years to be commercially available, with a success rate of only 2.01% (Yeu et al., 2015; Xue et al., 2018). Not to mention that nearly 90% of all clinical drug development fails due to the absence of clinical efficacy, unmanageable toxicity, poor drug-like properties, and lack of commercial demand (Sun et al., 2022). Alternatively, drug repurposing (DR, also drug repositioning, redirecting, reprofiling, and re-tasking), which consists of using existing drugs for new disease indications, can hasten the drug discovery process through the use of validated, toxicologically safe, and already regulated drugs (Martínez-García and Hernandez-Lemus, 2021), making DR a hot topic, currently.

Many methodologies can be used to achieve DR and overcome the difficulties and lengthy processes inherent to new drug discovery. An efficient DR workflow for both Academia and pharmaceutical companies requires the combination of availability and access to molecular data, analytical expertise provided by a cross-disciplinary team, experimental set-up for validation, and clinical development process (Cha et al., 2018). In this regard, repurposing methods can be subtly divided into those based on experimental screening settings and *in silico* approaches that analyze existing data to identify potential new drug-disease associations.

Although we may have witnessed much progress in the field, the gap between what can be found through computational methodologies and drug repurposing itself is yet to be fully understood. As a matter of fact, repurposing is successful only in certain cases. It denotes that these computational approaches have been misleading, primarily because of the questionable chosen input data, poor data quality, and debatable analysis methods. In addition, patent issues, regulatory considerations, and organizational hurdles also make drug repurposing hard to be implemented (Pushpakom et al., 2018). Thus, this review aims to give an overview of the general approaches to computational drug repurposing, showcasing the gap between the *in silico* strategies and their practical application in drug repurposing in oncology.

## 2 Material and methods

### 2.1 Study protocol and registration

This scoping review followed the methodological framework proposed by Arksey and O’Malley (2005) and complies with the recommendations of the Joanna Briggs Institute for elaborating scoping reviews (Peters et al., 2015). The protocol has been previously published (Cavalcante et al., 2022) and registered on the Open Science Framework (https://osf.io/yx7kp). Additionally, this review is reported following the PRISMA extension for scoping reviews (Supplementary File S1) (Moher et al., 2015; Page et al., 2021).

### 2.2 Population, concept, and context

We used the population-concept-context (PCC) mnemonic to guide the research questions, eligibility criteria, and literature search. The articles in this scoping review should focus on *in silico* approaches for drug repurposing (concept) within the Oncology research field (context). We did not specify a population since we were interested in computational methods used in Oncology research in general, including different study designs and populations (Table 1).

**TABLE 1:** The main population-concept-context mnemonic (PCC).

Population	Concept	Context
N/A	The main concept of interest is *in silico* approaches for drug repurposing	Oncology field

### 2.3 Research question

The following research question guided this scoping review: How can *in silico* strategies be implemented in DR in oncology? Based on this question, the three main objectives were: 1) to identify the most used *in silico* strategies, 2) to identify the gap between what can be found through computational strategies and the lacking knowledge concerning DR in oncology and 3) to identify the regulatory barriers to DR when using *in silico* strategies in cancer research.

### 2.4 Eligibility criteria

The eligibility criteria were determined through the PCC mnemonic. We included peer-reviewed, English-language research articles published between January 2003 and December 2021 that have implemented *in silico* strategies for DR in oncology. We excluded narrative or systematic reviews, book chapters, author’s opinions/comments, editorials, erratum, meeting abstracts, conference abstracts, and study protocols. Also, we excluded studies that used *in silico* strategies for other objectives rather than DR, where no abstract was available or full-text articles could not be obtained, and studies on DR in oncology intended for animal use.

### 2.5 Information sources and search

At first, a preliminary search was conducted on Pubmed (Medline) to map relevant articles on DR in oncology. The titles and abstracts of these articles and their keywords were used to create a comprehensive search strategy. Our search strategy included Medical Subject Headings (MeSH) terms, their corresponding synonyms, and the Boolean operators “AND” and “OR”. The search strategy was organized into three concept clusters: 1) *in silico* approach, 2) drug repurposing, and 3) oncology. The search strategy initially created for Pubmed (Medline) (Table 2) was modified for the Embase, Scopus, and Web of Science databases. We also explored the grey literature through Open Grey, and the reference lists of all the included sources of evidence were examined to identify any additional studies (Supplementary File S2). The electronic search strategies were conducted on 22 March 2022. Details regarding the search strategies employed in each database were previously published (Cavalcante et al., 2022).

**TABLE 2:** Search strategy for Pubmed (Medline).

Database	Search strategy
PubMed	[(“drug repositioning” OR “drug repurposing” OR “drug rescue” OR “off-label use” OR “off-label uses” OR “off-label prescribing” OR “unlabeled indication” OR “high throughput screening” OR “high throughput screening assays”) AND (“*in silico*” OR “in silicos” OR “computer simulation” OR “computerized model") AND (oncolog* OR cancer* OR tumor* OR tumour* OR neoplas*)]

### 2.6 Selection of sources of evidence

Following the search, all identified citations were exported in Comma-Separated Values (CSV) format to Microsoft Excel (Version 2019), and duplicates were removed. Two independent reviewers (BRRC and LOSR) screened all titles and abstracts for assessment against the inclusion criteria. The full text of selected articles was assessed against the exclusion criteria by the same reviewers, independently. Any disagreements between the reviewers were resolved through discussion. The inter-rater agreement was assessed through Cohen’s κ before the abstract review stage using the Jamovi software (version 1.2).

### 2.7 Data charting and data items

Data was extracted from the included articles using a Microsoft Excel spreadsheet (Version 2019). Following the review questions, two independent reviewers (BRRC and GVR) developed and tested the data charting tool with ten random studies. Data extracted comprised the following items: 1) title, 2) publication date, 3) authors, 4) country, 5) study aim, 6) study design, 7) type of cancer, 8) *in silico* method used, 9) study outcome, 10) mention of regulatory aspects, and 11) occurrence of drug rescue or repurposing.

### 2.8 Synthesis of results

We present the results narratively and use tables containing the topics detailed in the previous section. The key findings are described according to the review questions, with maps to portray the geographical location of publications and graphs for better data visualization. Also, this review was divided into sections that include the main results of individual sources of evidence followed by the most used methodologies in the included studies and challenges in DR in oncology.

The world map was constructed using the rnaturalearth and ggplot2 packages in the R programming environment (version 4.3.1). These packages provided the geographical data of the globe, where we built a data frame of the publication count with the selected countries from the previous charting of the studied articles. Publications that involved the collaboration of different countries, each country was counted as one. Next, we merged the map with the data frame and generated the plot using the geom_sf function to plot the selected countries. We also used the scale_fill_gradient function to create a color scale for the assigned values.

## 3 Results

### 3.1 Selection of sources of evidence

**FIGURE 1:**
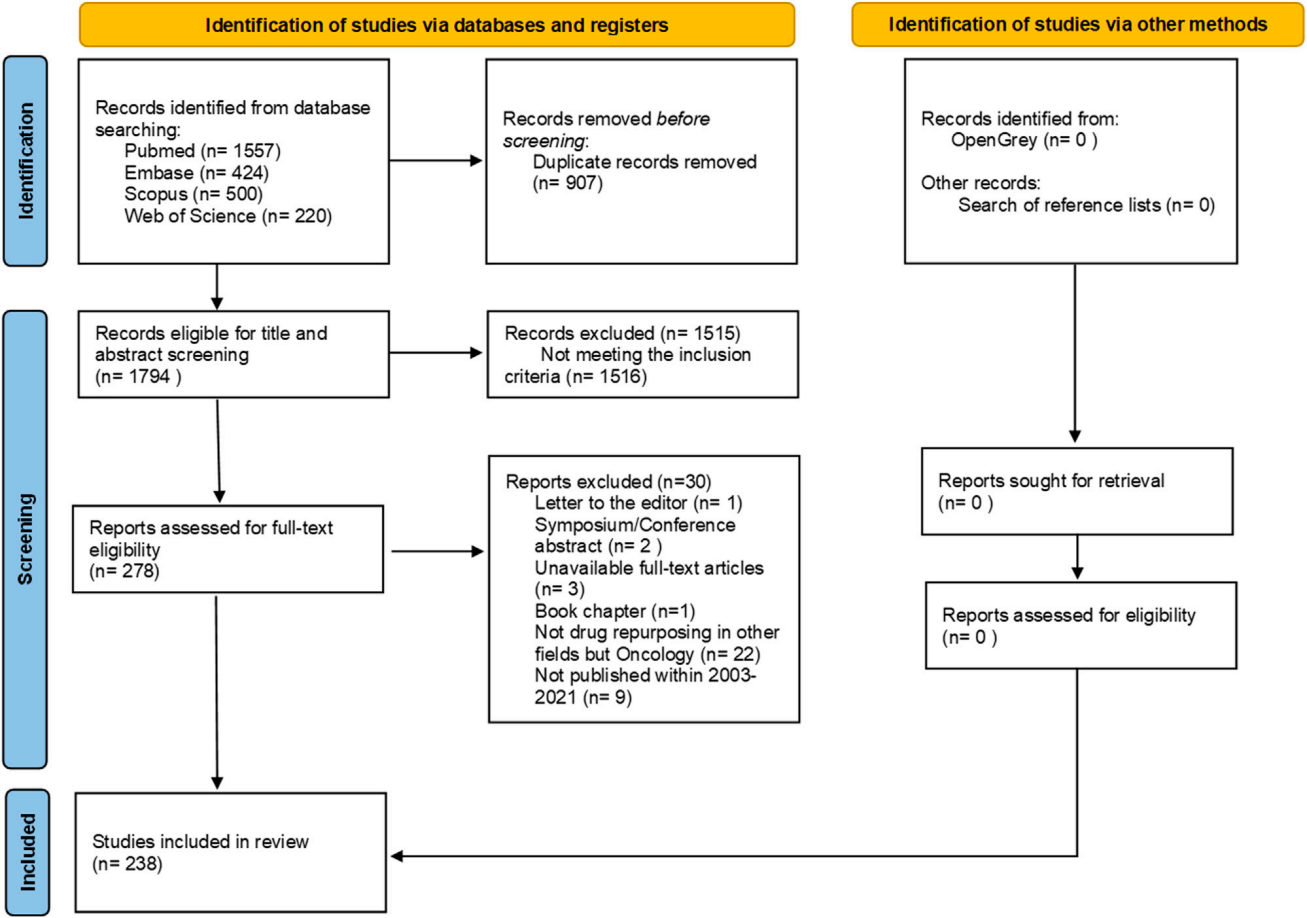
Flow diagram of literature search and selection criteria adapted from PRISMA (Page et al., 2021).

We identified 2,701 articles through our search, 907 of which were duplicates. After de-duplicating the sample, we screened 1,794 articles for relevance. The full list of records screened for eligibility are available at OSF (https://osf.io/yx7kp/files). We found 1,515 records eligible for full-text review, 279 of which were included in our complete analysis. After thorough analysis, 41 articles were excluded for not meeting inclusion criteria, leaving 238 articles eligible for full-text scrutiny (Figure 1). A table with the summary of all included studies is available in Supplementary File S3 and at OSF (See “data charting” at https://osf.io/yx7kp/files/).

### 3.2 Characteristics of sources of evidence

**FIGURE 2:**
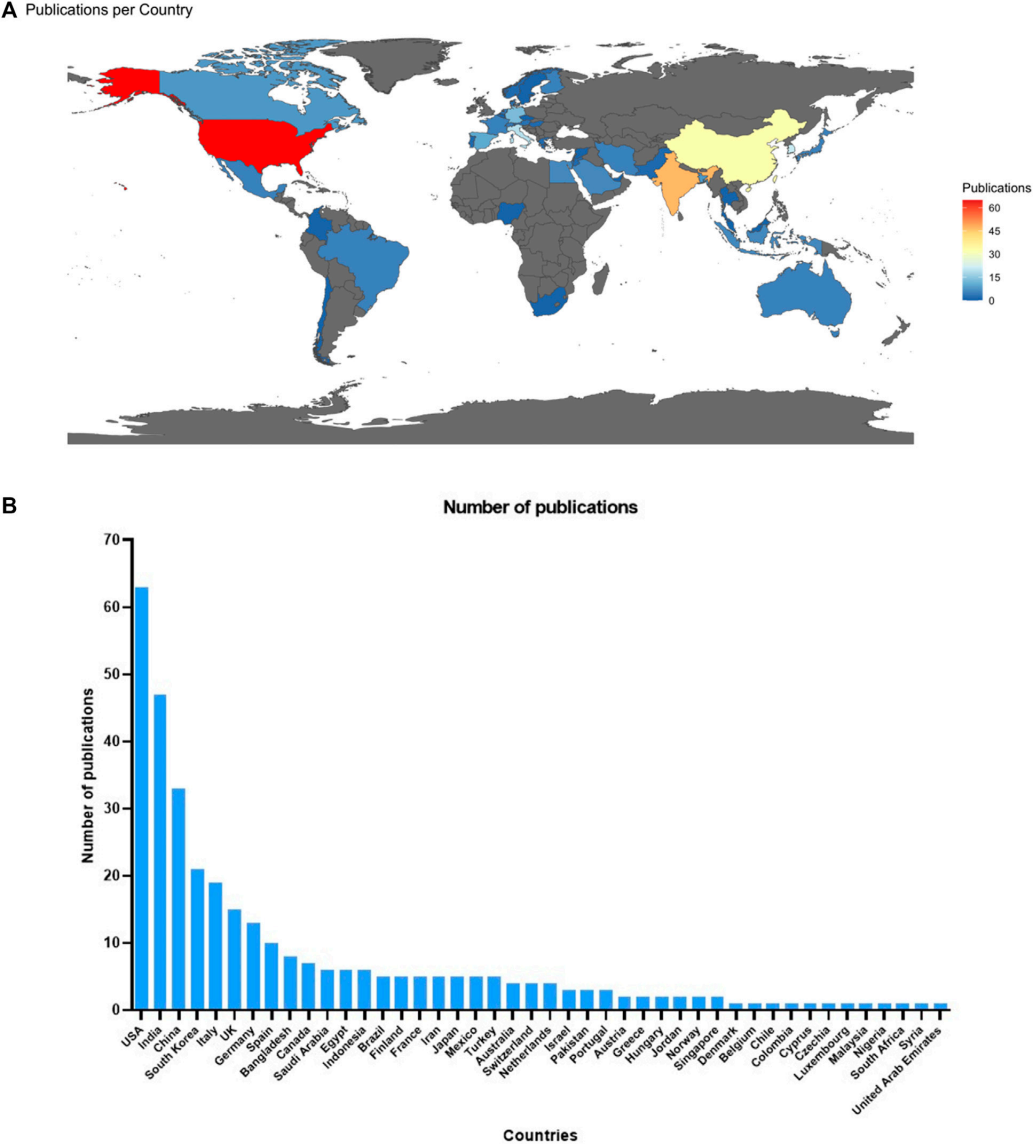
**(A)** Worldwide distribution of selected studies about drug repurposing in oncology (*n* = 238). **(B)** Distribution of publications per country.

The 238 studies that met our inclusion criteria were developed in 44 countries (Figures 2A, B). Most studies identified were from the United States, India, China, South Korea, and Italy, which stand out as the top publishers. Other countries and regions, such as the United Kingdom, Germany, and Spain, have also contributed to the progress of employing computational approaches in oncology. The remaining countries had fewer than ten published studies across all the continents. The central-eastern portions of Europe, many regions of South America and Africa, and northern parts of the Asian continent are regions that did not appear in the data charting of the selected articles.

### 3.3 Results of individual sources of evidence and data synthesis

**FIGURE 3:**
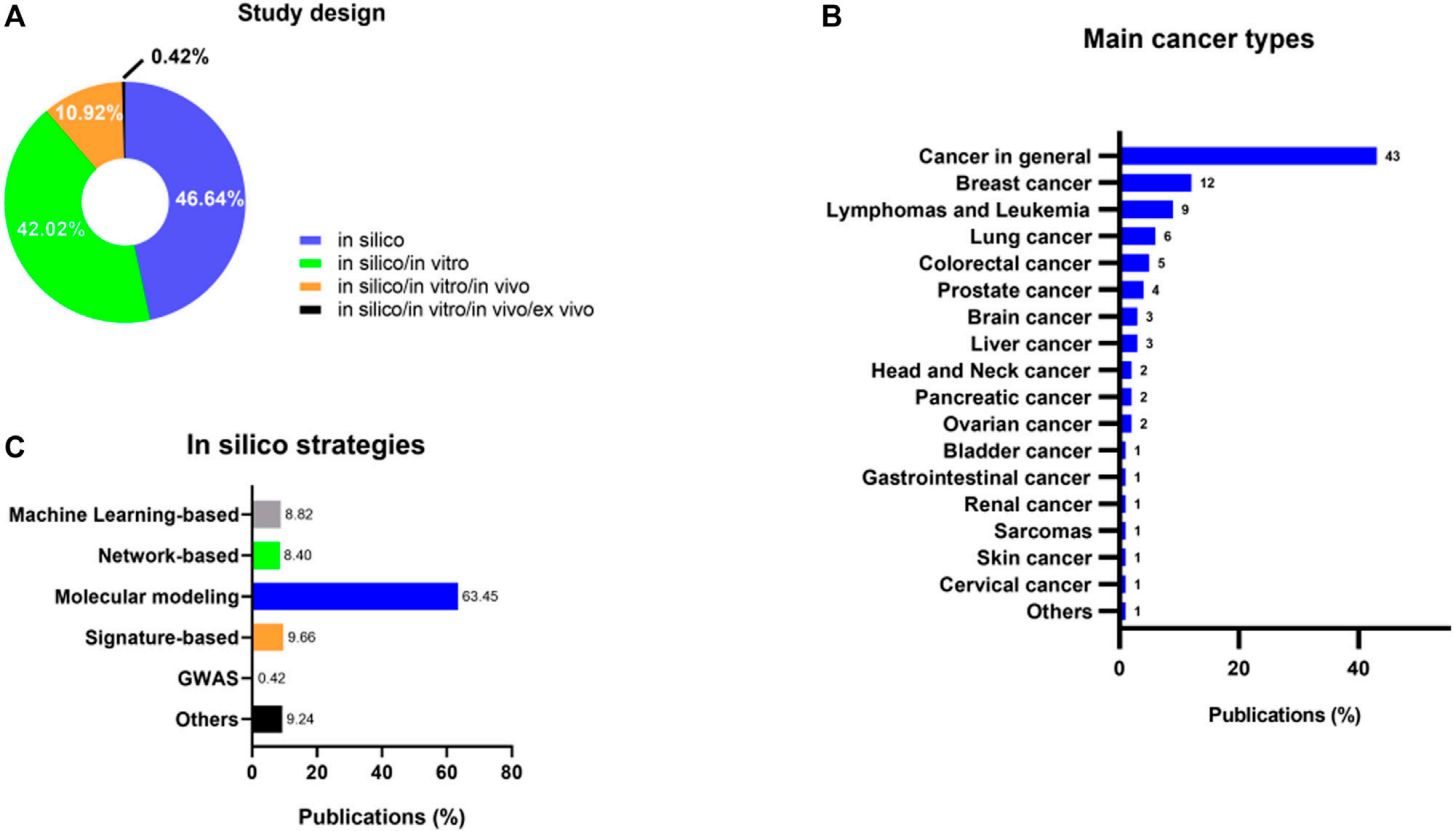
**(A)** Study design of publications broadly categorized as *in silico*, *in silico*/*in vitro*, *in silico*/*in vitro*/*in vivo*, and *in silico*/*in vitro*/*in vivo*/*ex vivo* models. **(B)** Prevalence of the most investigated cancer types under *in silico* DR approaches. **(C)** Prevalence of the most used methodologies in the included studies.

Regarding study design, we sought to investigate if the publications fit into a broad categorization of the *in silico* strategies. We found that most studies solely used computational methods without further testing their findings in other scientific settings (46.64%) (Shah et al., 2012; Abazeed et al., 2013; Ain et al., 2013; Eichhorn et al., 2013; Emig et al., 2013; Leung et al., 2013; Liu et al., 2013, 2020, 2021; Menden et al., 2013; Naresh et al., 2013; Omotuyi et al., 2013; Singh et al., 2013; Chen, 2014; Dean et al., 2014; Xu and Wang, 2014; Chuang et al., 2015; Dunna et al., 2015; Gustafson et al., 2015; Hammad and Azam, 2015; Kaur et al., 2015; Paula et al., 2015; Rubio-Perez et al., 2015; Sarvagalla et al., 2015; Zhang et al., 2015; Brown et al., 2016; Gayvert et al., 2016; Lee et al., 2016; Shafique et al., 2016; Tan, 2016; Verma et al., 2016; Spiliotopoulos et al., 2017a, 2017b; Jadhav and Karuppayil, 2017; Kabir et al., 2017, 2018, 2019; Khanam et al., 2017; Salentin et al., 2017; Sohraby et al., 2017; Ulfa et al., 2017; Arora and Singh, 2018; Barua et al., 2018; Fröhlich et al., 2018; Jubie et al., 2018; Juritz et al., 2018; Karube et al., 2018; Konidala et al., 2018; Lagarde et al., 2018; Mady et al., 2018; Montes-Grajales et al., 2018; Shi et al., 2018; Shin et al., 2018; Wang et al., 2018; Xu et al., 2018; Yu et al., 2018; Amusengeri and Bishop, 2019; Catalano et al., 2019; Cheng et al., 2019; Floresta et al., 2019; Kuthuru et al., 2019; Natarajan et al., 2019; Patidar et al., 2019; Ramos et al., 2019; Sahrawat and Kaur, 2019; Sweta et al., 2019; Turanli et al., 2019; Yadav et al., 2019; Yang et al., 2019, 2021; Çınaroğlu and Timuçin, 2019; Wu et al., 2020a; Choi et al., 2020; Jiang et al., 2020; Karthik and Vijayakumar, 2020; Kumar et al., 2020; Liñares-Blanco et al., 2020; Prakash and Nath Dwivedi, 2020; Shehadi et al., 2020; Siam et al., 2020; Aier et al., 2021a; Alisha and Tripti, 2021; Aier et al., 2021b; Biswas et al., 2021; Bourdakou et al., 2021; Cadow et al., 2021; Chandel et al., 2021; Florio et al., 2021; Kadioglu et al., 2021; Nasiri et al., 2021; Parveen, 2021; Radaeva et al., 2021; Saranyadevi, 2021; Song et al., 2021; Zagidullin et al., 2021; Deokar and Shaikh, 2022; Gao et al., 2022; Madhukar and Subbarao, 2022; Cell et al., 2024). Surprisingly, several studies combined *in silico* and *in vitro* approaches to validate their results (42.02%) (Jordheim et al., 2013; Lafleur et al., 2013; Schonbrunn et al., 2013; Tabchy et al., 2013; Brewer et al., 2014; Kanematsu et al., 2014; Stachnik et al., 2014; Takeuchi et al., 2014; Calvete et al., 2015; De La Cruz-Hernandez et al., 2015; Fako et al., 2015; Istyastono et al., 2015; Liao et al., 2015; McNeil et al., 2015; Rameseder et al., 2015; Riddick et al., 2015; Segura-Cabrera et al., 2015; Zalloum et al., 2015; Zeino et al., 2015; Zhong et al., 2015; Chong et al., 2016; Hengel et al., 2016; Lara-Castillo et al., 2016; Montes-Grajales et al., 2016; Radi et al., 2016; Rocca et al., 2016; Wilson et al., 2016; Chen et al., 2017; Di Lello et al., 2017; Digiacomo et al., 2017; Jaeger et al., 2017; Jernigan et al., 2017; Klingenberg et al., 2017; Kolosenko et al., 2017; Mould et al., 2017; Ozsvari et al., 2017; Platonova et al., 2017; Prabhu et al., 2017; Salim et al., 2017; Vilaboa et al., 2017; Yang et al., 2017; Amaral et al., 2018; Chang et al., 2018; He et al., 2018; Holt et al., 2018; Hyter et al., 2018; Krishna et al., 2018; Manohar et al., 2018; Ayoub et al., 2018; Sakr et al., 2018; Tian et al., 2018; Amrutha et al., 2019; Fischer et al., 2019; Li et al., 2019; Li et al., 2021; Metz et al., 2019; Mishra et al., 2019; Poli et al., 2019; Qi et al., 2019; Song et al., 2019; Ben-Hamo et al., 2019; Frejno et al., 2020; Gibbons et al., 2020; Anderson et al., 2020; Ariey-Bonnet et al., 2020; Naeem et al., 2020; Aydin et al., 2020; Strätker et al., 2020; Faridi et al., 2020; Velázquez-Quesada et al., 2020; Wakai et al., 2020; Benavides-Serrato et al., 2020; Wu et al., 2020b; Zhang J. et al., 2020; Bonnet et al., 2020; Al-Shar’i et al., 2021; Correia et al., 2021; Elkamhawy et al., 2021; Enríquez-Flores et al., 2021; Giubilaro et al., 2021; Nagamalla and Kumar, 2021; Parcha et al., 2021; Rashidieh et al., 2021; Renna et al., 2021; Rimpelová et al., 2021; Suphavilai et al., 2021; Traylor et al., 2021). Only a few publications combined *in silico*, *in vitro*, and *in vivo* methods (10.92%) (Chan et al., 2013; Shi et al., 2013; Van Kesteren et al., 2013; Choi D. S. et al., 2014, Choi et al., 2014 B. H.; Gazzard et al., 2014; Nakamura et al., 2014; Parks et al., 2014; Liu et al., 2015; Druzhyna et al., 2016; Jiang et al., 2017; Ke et al., 2017; Mirza et al., 2017; Pessetto et al., 2017; Kang et al., 2018; Lodagekar et al., 2019; Mottini et al., 2019; Zou et al., 2019; Zhang M. et al., 2020; Kwon et al., 2020; Chequin et al., 2021; Rabben et al., 2021; Sulsenti et al., 2021; Zhao et al., 2021), and a single study added *ex vivo* models in their approach (0.42%) (Jahchan et al., 2013) (Figure 3A).

Many cancer types can benefit from DR. While the majority of publications (43%) utilized computational techniques to target cancer broadly, including identifying inhibitors (Brewer et al., 2014; Zhong et al., 2015; Shafique et al., 2016; Ulfa et al., 2017; Çınaroğlu and Timuçin, 2019; Siam et al., 2020), agonists (Kaur et al., 2015; Wang et al., 2018), Drug-Target interaction predictions (Emig et al., 2013; Kuthuru et al., 2019), and virtual screening (Rocca et al., 2016; Spiliotopoulos et al., 2017b; Lagarde et al., 2018), a portion of the literature concentrated on specific cancer types. Among these, breast cancer (12%) (Emig et al., 2013; Jiang et al., 2017; Montes-Grajales et al., 2018; Velázquez-Quesada et al., 2020; Chequin et al., 2021), lymphomas and leukemias (9%) (Lara-Castillo et al., 2016; Sohraby et al., 2017; Karube et al., 2018; Sahrawat and Kaur, 2019; Boulos et al., 2021; Parcha et al., 2021), lung (6%) (Kwon et al., 2020; Saranyadevi, 2021), colorectal (5%) (Liñares-Blanco et al., 2020; Biswas et al., 2021; Deokar and Shaikh, 2022; Leung et al., 2022), and prostate (4%) (Kang et al., 2018; Ariey-Bonnet et al., 2020; Benavides-Serrato et al., 2020; Liu et al., 2021; Lin et al., 2022) cancers were the most frequently investigated (Figure 3B).

Several promising computational techniques have been developed to aid in repurposing. They can be broadly categorized as 1) signature-based, 2) molecular modeling, 3) network-based, 4) genome-wide association studies (GWAS) (Altay et al., 2020), and Machine Learning (ML)-based. Most of these methodologies require precise and curated data inputs that demand many datasets and databases. As shown in Figure 3C, molecular modeling (MM), which includes molecular docking (MD) and molecular dynamics simulations (MDS), has emerged as one of the most frequently used methods (63.45% of publications), followed by signature-based (9.66%). Next, some authors have employed ML- (8.82%) and network-based strategies (8.40%). Lastly, a single publication (0.42%) used GWAS to achieve DR. Supplementary File S4 summarizes the main *in silico* methods used for drug repurposing and their respective softwares.

### 3.4 Molecular modeling (molecular docking and molecular dynamics simulations)

In molecular modeling, MD has become an increasingly important tool for DR. It can be used to model the interaction between a small molecule and a protein at the atomic level (Meng et al., 2011). Then, some calculations can be performed to investigate the binding affinity of the ligands within the protein active site (i.e., groove or pocket formed by the folding pattern of the protein), elucidating fundamental biochemical processes. Complementarily, MDS can be used to obtain more accurate results since the dynamic behavior of proteins and other biomolecules can be monitored in full atomic detail and at different timescales since MDS allow the understanding of several critical biomolecular processes, such as conformational change, ligand binding, and protein folding, revealing the positions of all the atoms at femtosecond (Hollingsworth and Dror, 2018).

Some publications have used molecular modeling to identify new inhibitors (Mady et al., 2018; Radaeva et al., 2021) or propose new pipelines (Liu et al., 2020; Deokar and Shaikh, 2022), considering only *in silico* approaches. Nevertheless, some authors used MD as a kick-off start of a more extensive approach to check biological activity *in vitro* and *in vivo* for breast cancer and bladder carcinoma. Chequin et al. (2021) showed a repurposing strategy through molecular docking studies of the antitumoral activity of liraglutide, a well-known diabetes type II drug, by modulating epigenetic modifications in breast cancer cell lines *in vitro* and *in vivo* using Ehrlich mice tumors models. Ke et al. (2017) used MD to identify potential inhibitors of FGFR3 from 3,167 worldwide approved small-molecule drugs through a repositioning approach. After *in vitro* testing, the acaricide drug fluazuron exhibited the highest anti-proliferative effect in human bladder carcinoma cell lines RT112 and RT4 and anticancer effect *in vivo* in BALB/C nude mice subcutaneously xenografted with RT112, suggesting that fluazuron is a potential inhibitor of FGFR3 and a candidate anticancer drug for the treatment of this carcinoma.

Some authors have taken advantage of molecular studies to elucidate whether some drugs display relevant binding properties. Kang et al. (2018) used Molecular modeling simulation to explain the capability of the synthesized analogs to increase the intracellular Ca^2+^ levels to understand variations in the ability of synthesized analogs of the antipsychotic drug trifluoperazine for the treatment of glioblastoma. They identified fourteen compounds that were biologically active *in vitro* and *in vivo* (assessed in brain xenograft mouse model of glioblastoma), presenting the compound 3dc as a new tool for the adjuvant chemotherapy of glioblastoma. Druzhyna et al. (2016) used docking simulations to elucidate how benserazide, a DOPA decarboxylase inhibitor, is related to the inhibition of cystathionine-β-synthase activity and suppresses colon cancer cell proliferation and bioenergetics *in vitro* and tumor growth *in vivo* in nude mice bearing human colon cancer cell.

Although applications of combined workflows, including MD and MDS, have been explored to assist different tasks of DR, it is noteworthy to mention that each computational method has its limitations, especially when analyzing less-characterized molecular targets (Pinzi and Rastelli, 2019).

### 3.5 Signature-based strategies

The exponential growth of omics data across multiple biological levels, encompassing genomics, transcriptomics, and metabolomics, has presented significant opportunities for identifying previously undisclosed targets and comprehending underlying mechanisms in various cancer types. The accessibility of such data in publicly accessible repositories has played an indispensable role in driving substantial progress within the realm of DR research, particularly in exploring genetic signatures associated with dysfunctional signaling pathways in cancer. Signature-based methods are frequently employed to compare gene expression profiles between non-disease and disease states to identify specific gene signatures, which can serve as targets for repurposing existing drugs or guiding the development of new therapeutic compounds. This approach holds the potential for more targeted and effective therapeutic strategies in treating diseases.

Choi D. S. et al. (2014) identified Chloroquine as a potential cancer stem cell (CSC) inhibitor through *in silico* gene expression signature analysis of the CD44(+)/CD24(-/low) CSC population in Triple-negative breast cancer (TNBC). They reported that Chloroquine could sensitize TNBC cells to paclitaxel through inhibition of autophagy and reduced the CD44(+)/CD24(-/low) CSC population in both preclinical and clinical settings. Similarly, to identify new therapeutic options for Ewing sarcoma (EWS), Pessetto et al. (2017) employed an integrated bioinformatics approach based on disease signature alongside an *in vitro* screen of FDA-approved drugs to predict drug activity. They showed that two drugs, Auranofin (a thioredoxin reductase inhibitor) and Ganetespib (an HSP90 inhibitor), displayed anticancer activities *in silico* and *in vitro*. Still taking advantage of disease signature patterns, Jahchan et al. (2013) employed a DR strategy to identify FDA-approved candidate drugs to treat non-small cell lung cancer (SCLC). They identified tricyclic antidepressants and related G protein–coupled receptor inhibitors as potent inducers of cell death in SCLC cells and other neuroendocrine tumors.

Although widely employed for DR, signature-based approaches face significant challenges. There are genetic signatures that do not provide comprehensive coverage of the transcriptome, that is, all possible gene expressions in an organism. Also, interpreting these signatures often requires a deep understanding of the biological context and the intricate interactions between genes and proteins, which may hinder the identification of causal relationships between signatures and observed effects.

### 3.6 Machine learning-based approaches

ML is a branch of artificial intelligence (AI) and refers to acquiring predictive information or identifying informative clusters within data. In recent years, ML has been in the spotlight, being applied to several purposes, including the field of drug design, such as the prediction of drug–target interaction and drug discovery. In fact, ML has been used in many other approaches revised in this work, including structure-based approximations (ML-based scoring functions), network-based approaches, etc. Overall, machine learning offers powerful tools and methodologies for accelerating drug repurposing efforts, enabling researchers to leverage existing knowledge and data to identify new therapeutic opportunities, improve drug safety and efficacy, and advance personalized medicine approaches.

Since DR depends on extensive data from existing drugs and diseases, ML methods have been widely used to supply the application of data science to signaling disease, medicine, therapeutics, and identifying potential targets (Yang et al., 2022). Song et al. (2021) developed an ML approach to infer unknown DTIs for breast cancer, called PsePDC-DTIs. The model achieved good prediction results and provided ten potential DTIs. Further, Salim et al. (2017) identified the molecule COTI-2 through a computational platform and checked its role in *in vitro* and *in vivo* settings. They showed that COTI-2 was effective against a diverse group of human cancer cell lines regardless of their tissue of origin or genetic makeup. Anderson et al. (2020) used two strategies to enable DR, one by taking advantage of ML models of chordoma inhibition to further screen compounds of interest *in vitro* and the other by testing combinations of approved kinase inhibitors already being evaluated for chordoma.

If, on one hand, ML has brought more understanding and has provided advances in DR, on the other hand, some limitations may pose some challenges for its implementation, which involve (a) the lack or quality of good data, (b) bias and discrimination (the data might not represent overall population), (c) overfitting and underfitting problems (when ML models fail to provide a suitable output), and (d) lack of reproducibility (inability to replicate or reproduce the results of a ML experiment or study).

### 3.7 Network-based approaches

Network-based approaches have emerged as promising methods for DR, specifically for treating diverse types of cancer. These techniques require the integration of multiple data sources, such as publicly available datasets and high-throughput data, to model the molecular interactions within complex biological systems using graph theory. In this system, the nodes can be represented by drugs, proteins, diseases, or genes, and edges represent their relationships. The networks modeling drug-drug, drug-target, drug-disease, disease-gene, and protein-protein interactions can provide insights into non-obvious associations related to drug mechanisms of action (MOA).

Since many drug targets act like transcription factors in gene regulation, transcriptome data are extensively used to identify potential drug targets through gene regulatory networks. Network measures such as neighborhood scoring, interconnectivity, network propagation, and random walk enable the discovery of new gene-target associations, prioritizing new candidate targets, and revealing new diseases associated with the selected target.

Protein-protein interaction (PPI) networks are strategies to find drug targets that interact directly or indirectly with other proteins. This method aims to predict drug-target interactions (DTI), considering that proteins targeted by similar drugs are neighbors and functionally related within the interaction network. Many drugs are non-specific and may modulate additional targets beyond their primary targets. Therefore, in addition to protein-protein similarity, network analysis is employed to elucidate drug-drug similarity. When a drug has a known interaction target, models can be predicted based on protein similarity; however, if the drug lacks a known target, its potential target is determined based on the similarity of its molecular structure. Moreover, proteins exhibiting high sequence similarity may interact with analogous drugs.

Some authors used network analysis to provide insightful strategies to promote DR. Emig et al. (2013), for instance, employed a network-based approach for the prediction of drug targets for different types of cancer with their underlying biological processes by using cancer gene expression signature and a high-quality interaction network as inputs and having a prioritized list of drug targets as outputs. Jaeger et al. (2017) used a computational network biology approach to discover new synergistic drug combinations for breast cancer treatment and many cancer types. They found that the combination of raloxifene with the c-Met/VEGFR2 kinase inhibitor cabozantinib potentiated the drugs’ individual antitumor effects in a mouse model of breast cancer. Another exciting approach proposed by Segura-Cabrera et al. (2015) integrated random walk-based network framework to identify known and novel drug indications against different subsets of breast cancers through contextual prioritization based on genome-wide gene expression, shRNA, and drug screen and clinical survival data, conceiving a platform, NetWalker (http://netwalkersuite.org), for contextual prioritization of drugs, genes, and pathways. Similarly, Cheng et al. (2019) proposed an integrated, network-based methodology for cancer type-specific disease module identification and *in silico* drug repurposing. They developed a Genome-Wide Positioning System Network (GPSnet) algorithm for DR using whole-exome sequencing and transcriptome profiles from ∼5,000 patients across 15 cancer types from The Cancer Genome Atlas. They found that ouabain, an approved cardiac arrhythmia and heart failure drug, displays potential antitumor activities in lung adenocarcinoma.

The drug repositioning strategies based on applying molecular interactomes have significant limitations. There needs to be a complete understanding of the molecular interactions at different biological levels: the interactions among the various molecules within a cell or tissue can encompass interactions between different levels, from the interaction of two proteins to the interaction of multiple metabolic pathways. This complexity requires the consideration of many variables that affect molecular interactions, which can lead to inconsistent or difficult-to-interpret results.

### 3.8 Genome-wide association studies

Current scientific advances have allowed new visions and therapeutic strategies to be included in medical approaches, making room for an increasingly personalized and inclusive medicine regarding human variability. In this context, one relevant approach is the GWAS.

GWAS comprise a genomic approach that uses the association of genotypes with the phenotypes by testing for differences in the allele frequency of genetic variants between individuals who are ancestrally similar but differ phenotypically (Uffelmann et al., 2021). In this approach, DNA strand *loci* are used for further analysis of the allele frequency of this region. GWAS aims to identify genetic variants, or single nucleotide polymorphisms (SNPs), that are associated with a particular trait or disease. These SNPs are then used to create a genomic risk score that can be used to predict an individual’s likelihood of developing the disease or trait.

Over the past decades, GWAS have uncovered many genetic variants that may provide targets for DR. However, only one publication used GWAS to promote DR. Zhang et al. (2015) developed an *in silico* pipeline based on GWAS to prioritize the candidate genes at the colorectal cancer risk *loci*, targeting them with approved therapies for colorectal cancer, such as crizotinib, arsenic trioxide, vrinostat, dasatinib, estramustine, and tamibarotene.

The valuable information GWAS provide has outstanding potential to guide drug discovery or repurposing. However, there are several limitations underlying these studies, which include: (a) the top genes identified from GWAS may not be easily druggable, (b) the focus on the effect of the top SNPs may miss biologically relevant target genes with small effect sizes, (c) the use of a single candidate gene may miss multi-target drugs, and (d) the inadequate annotation procedures due to the complexity of the human genome (Lau and So, 2020).

### 3.9 Why is it so challenging to use computational strategies to promote *de facto* drug repurposing in oncology?

Cancer is a complex disease encompassing a range of different tumor types in terms of genetic and molecular characteristics (Shlyakhtina et al., 2021; Hanahan, 2022) and demands new therapeutic agents. Although DR through computational approaches might be beneficial, it has been quite challenging to implement for numerous reasons.

Several public databases, such as The Cancer Genome Atlas Program (TCGA), broadly provided omics (e.g., genomic, transcriptomics, and proteomic) and clinical data, enabling comprehensive *in silico* studies in DR in oncology (Mottini et al., 2021). However, high-quality data is not always readily accessible or standardized across studies and databases (Grannis et al., 2019). For the results to be reliable, the datasets must be accurate, comprehensive, reproducible, and representative. Data quality is key for any scientific and clinical research, and it starts with mitigating data imprecisions that lead to inaccurate or misleading analytics results. Some problems include unstructured dataset metadata and inconsistent data processing and quality control, which demand crucial extensive curation and data reprocessing (Lim et al., 2021). Due to the data’s heterogeneity, combining different data types, such as transcriptomic data, chemical structure data, and clinical literature data, poses another computational challenge for effective DR (Ko, 2020). Additionally, relevant and public patient data is limited in low- and mid-income countries (Hung et al., 2020; Abdul-Rahman et al., 2023). Other issues involve reproducibility and replicability in science. Although there are journals with an open data and code policy that require that, as a rule, code and data be released at the latest upon publication (Laurinavichyute et al., 2022), several published studies do not supply raw data and codes, leading to a high burden for those conducting the reproductions (Seibold et al., 2021). Underrepresented data in genomics and multi-omics studies, especially regarding racial and ethnic groups, has hindered advances in human genetics, with the vast majority of research conducted, developed, and validated in individuals of European descent (Landry et al., 2018).

Translating computational predictions into experimental preclinical validation and clinical studies requires time, resources, and collaboration among researchers, clinicians, and regulatory agencies (Seyhan, 2019). Regarding cancer, capturing the complexity and dynamics of the microenvironment (Baghban et al., 2020) through computational strategies, cell lines, and animal models struggles to predict drug responses accurately. Thus, identifying existing drug candidates for repurposing requires a deep understanding of underlying biological mechanisms and pathways involved in diverse cancer types. The accurate prediction of potential drug candidates and their efficacy is related to the knowledge of drug mechanisms, potential side effects, and safety concerns (Berlin et al., 2008). However, there are numerous drugs whose mechanisms and targets still need to be fully understood, and some drugs may have multiple targets or display off-target effects (Palve et al., 2021), further complicating the repurposing process.

The gap between *in silico* approaches and DR arises because computational strategies and experimental studies may sometimes disagree. The simulations’ results must be validated through experimental and clinical studies before they can be used to support drug repurposing efforts. *In vitro* and *in vivo* models are commonly used to validate the predicted potential drugs. Nevertheless, these settings diverge from physiological conditions, requiring new robust experimental designs that resemble *in vivo* tissue and disease pathology (Ko, 2020), which can be costly. Since drug response differs among patients, more clinical studies are needed to provide a new appropriate dose for the candidate drug since the original dose might no longer be the same. Indeed, pharmaceutical companies still struggle with rising clinical trial costs yearly due to more complex clinical development programs, which involve increased regulatory scrutiny and the growing need to demonstrate the safety and efficacy of new drugs and their value (Martin et al., 2017). A recent paper that assessed the clinical trials cost, gathering data from 726 studies conducted in patients from 2010 to 2015, showed that the median cost of conducting a study from protocol approval to final clinical trial report was US$3.4 million for phase I trials involving patients, $8.6 million for phase II trials and $21.4 million for phase III trials (Martin et al., 2017). Although repurposing a drug can be a cost-effective alternative to developing a new drug, the cost of clinical trials and regulatory submissions can still be substantial.

### 3.10 Regulatory barriers to drug repurposing when using *in silico* strategies in cancer research

DR can face several difficulties in the regulatory agencies’ approval process. Overcoming the legal and regulatory barriers is critical for succeeding, as the potential drug candidates must comply with regulatory agencies’ demands. In an ideal scenario, those drugs that show relevant clinical endpoints must be available quickly. In this regard, the US Food and Drug Administration (FDA) has an accelerated approval pathway for cases in which there is an improvement in overall survival or patients willing to tolerate uncertainty about such benefits in exchange for early access to promising cancer drugs (Gyawali et al., 2021). However, the same is not true regarding how other agencies worldwide deal with this issue. Considering that different countries may have different requirements for drug approval, it is difficult to obtain global approval for a repurposed drug. Overall, the approval process for DR can be more challenging than for new drugs. Still, with the increasing recognition of the potential benefits of drug repurposing, regulatory agencies are expected to become more willing to consider repurposed drugs for approval.

Intellectual property issues must also be considered concerning DR. Pharmaceutical companies may only be willing to invest in developing repurposed drugs if they can obtain new patents for the new indications (Krishnamurthy et al., 2022). Another problem is that pharmaceutical companies were described as patenting many compounds, even if they will be abandoned later, thus preventing others from developing these compounds without a license (Tartaglia, 2006). Overall, the financial support for DR has been insufficient, along with shorter patent durations and low returns on investments.

## 4 Discussion

Computational methods contribute to the process of drug repurposing by streamlining target discovery and drug screening at a preclinical level. This scoping review has highlighted significant advancements and challenges in the field of DR in oncology, led by a variety of *in silico* strategies. We have shown the dominance of certain countries, such as the United States, India, China, South Korea, and Italy, in computational approaches for drug repurposing for cancer treatment. Overall, these countries often have well-established research infrastructures, including academic institutions, research centers and collaborative networks, which enable the establishment of a thriving environment, filled with necessary resources and expertise for conducting computational research in drug repurposing. Additionally, these countries are often leaders in technological innovation, including advancements in artificial intelligence, machine learning, and bioinformatics. Finally, countries with large populations, such as India and China, may have access to diverse patient populations and clinical datasets, which are invaluable for training computational models and identifying potential drug candidates for repurposing.

Molecular modeling is crucial in drug repurposing efforts in oncology by providing insights into the interactions between drugs and their molecular targets. This can be achieved by predicting existing drugs’ binding affinity and mode of action against specific molecular targets implicated in cancer. Molecular modeling provides detailed structural insights into drug-target interactions and enables rational drug design, whereas signature-, machine learning-, and network-based strategies offer complementary approaches for identifying potential drug candidates and understanding drug-disease associations. These approaches lack mechanistic interpretability or correlations without explicit consideration of molecular structure and function. The incorporation of molecular modeling, particularly MD and MDS, has identified novel inhibitors and repurposing strategies for existing drugs, as evidenced in studies targeting breast cancer and bladder carcinoma (Ke et al., 2017; Chequin et al., 2021). However, applying these methodologies is limited, especially when exploring less-characterized molecular targets (Pinzi and Rastelli, 2019).

Signature-based strategies have capitalized on the rapid expansion of omics data, revealing new targets and disease mechanisms. These methods hold great promise in the quest for potential cancer treatments, as exemplified by the identification of Chloroquine for Triple-negative breast cancer (TNBC) and Auranofin and Ganetespib for Ewing sarcoma (EWS)) (Choi D. S. et al., 2014; Pessetto et al., 2017). However, the challenges associated with these strategies, such as the incomplete coverage of the transcriptome and the complexity of interpreting gene expression signatures within their biological context, remain (Jahchan et al., 2013).

ML-based approaches have introduced a new perspective to DR, enabling the prediction of drug-target interactions and the discovery of new drugs. Notable examples include creating the PsePDC-DTIs model for breast cancer and identifying COTI-2 as an effective agent against various cancer cell lines (Salim et al., 2017; Song et al., 2021). However, the effectiveness of ML models is dependent on the quality and representativeness of the data, and challenges such as bias, overfitting, and lack of reproducibility continue to exist (Yang et al., 2022).

Network-based approaches offer a holistic view of the molecular interactions in cancer, facilitating the discovery of new drug targets and drug-drug similarities. These methods have been successfully employed in predicting drug targets and synergistic drug combinations for various cancers (Emig et al., 2013; Jaeger et al., 2017). Nevertheless, the complexity of molecular interactions and the need for comprehensive data integration present significant hurdles (Segura-Cabrera et al., 2015).

GWAS have also contributed to DR, identifying genetic variants linked to specific diseases. Zhang et al.'s use of GWAS for colorectal cancer illustrates this approach’s potential (Zhang et al., 2015). Nevertheless, the challenges associated with GWAS, including the difficulty in identifying druggable genes and the limitations of focusing on single-gene effects, must be considered (Lau and So, 2020).

The overarching challenge in applying computational strategies for DR in oncology lies in the disease’s complexity and the discrepancies between *in silico* predictions and experimental validations (Shlyakhtina et al., 2021; Hanahan, 2022), since translating computational findings into clinical applications demands considerable resources and collaboration, with the additional complexity of capturing tumor microenvironments and predicting drug responses (Berlin et al., 2008; Baghban et al., 2020). However, our study identified many works that have employed solely computational approaches. Without further validations and tests, these computational findings become merely suggestive or predictive, hindering the chances of a potentially repurposed drug reaching clinical practice. Furthermore, repurposing drugs also implies that other scientific methodologies (i.e., *in vitro* validations, *in vivo* or *ex vivo* models, accurate toxicology studies, efficacy and effectiveness trials) must be undertaken to reduce the risks to the patient and increase the success rate of repurposed drugs in the product development process.

Lastly, regulatory barriers and intellectual property issues pose significant drug approval and commercialization challenges. The varied requirements of different regulatory agencies and the financial aspects associated with DR, including patenting and investment returns, are critical considerations in this field (Tartaglia, 2006; Gyawali et al., 2021; Krishnamurthy et al., 2022). Additionally, intellectual property issues and market considerations significantly impact the uptake and commercialization of repurposed drugs for cancer treatment. While patent protection and market exclusivity incentivize investment in repurposing efforts, they can also lead to pricing pressures and access barriers. Balancing the need for innovation and profitability with patient access and affordability is essential to ensure that repurposed drugs effectively address unmet medical needs in cancer treatment.

Computational methods for drug repurposing in cancer treatment have limitations that restrain their use to a preclinical level. Molecular models that predict compound-target interaction and treatment response may result in an oversimplification of the pharmacological complexity behind oncological chemotherapy, as it does not consider tumor heterogeneity, spatial and structural complexity, and potential treatment resistance development. Additionally, using biomedical databases harbors a potential human error, as information may be incomplete or misleading, resulting in output bias. Therefore, the application of *in silico* approaches in drug repurposing does not excuse the validation of results through qualified *in vitro* or *in vivo* methods. Finally, most datasets consist of transcriptomes from Caucasian populations, reflecting the lack of representation of minorities in clinical studies. In this sense, it is hard to extrapolate results and adapt promising treatments in Caucasian populations to a racially diverse population, especially black and Hispanic ethnic-racial identities.

Expediting drug discovery through repurposing existing medications for cancer treatment has ethical implications. This requires careful consideration of patient safety, transparency in communication, equitable access to treatments, robust regulatory oversight, management of conflicts of interest, and responsible stewardship of resources. Collaboration among stakeholders, including patients, healthcare providers, researchers, regulatory agencies, and pharmaceutical companies, is essential to navigate these ethical challenges and ensure that the benefits of expediting drug discovery through repurposing existing medications outweigh the potential risks.

As a scoping review, a notable limitation of this study design is the absence of a critical quality assessment of the included studies. However, it is crucial to emphasize that the objective of this review was to map the available evidence, rather than to conduct a methodological quality appraisal of the studies. For such detailed evaluations, more focused systematic reviews are advisable. Another limitation observed in this scoping review is that the studies generally failed to provide scripts or databases necessary for reproducibility. Furthermore, it is significant to point out the lack of established guidelines for a robust methodology or best practices in implementing *in silico* approaches, which should be explored in further research within this field.

## 5 Conclusion

Cancer is a generic term for a large group of diseases in which some are more likely to have a more suitable treatment and eventually be cured than others. Because each cancer needs to be treated differently, new approaches, including chemical entities, must be taken to improve oncological patients’ outcomes. In this scenario, repurposing drugs might be an advantageous alternative for faster clinical translation.

DR is an exciting opportunity to give the existing marketed drug new therapeutic indications. However, identifying potential drugs to be repurposed is not trivial, and implementing DR can be challenging due to various factors, including lack of quality data, patient populations, cost, intellectual property issues, market considerations, and regulatory requirements.

It is important to note that the trend of DR continues to grow, propelled mainly by employing advanced AI techniques. These techniques offer fresh perspectives on disease drug targets and enhance the likelihood of successful drug repurposing. In conclusion, despite the inherent challenges, DR is a promising strategy for discovering new treatments for a range of diseases, including various types of cancer, facilitating quicker patient access to novel medications and treatments.

## Data Availability

The original contributions presented in the study are included in the article/Supplementary Material, further inquiries can be directed to the corresponding author.
