# Supplementary material for: In silico approaches for drug repurposing in oncology: a scoping review
Source: Front Pharmacol. 2024 Jun 11;15:1400029. doi: 10.3389/fphar.2024.1400029 (PMC11196849; doi:10.3389/fphar.2024.1400029)
Supplement: Supplementary file 1 [file DataSheet2.PDF]

frontiers

Supplementary Material

Supplementary File 2. Search Strategy (March 22nd, 2022).

Database	Search	Results
**PubMed**	((“drug repositioning” OR “drug repurposing” OR “drug rescue” OR “off-label use” OR “off-label uses” OR “off-label prescribing” OR “unlabeled indication” OR "high throughput screening” OR "high throughput screening assays") AND (“in silico” OR “in silicos” OR “computer simulation” OR “computerized model”))	1557
**Embase**	((‘drug repositioning’/exp OR ‘drug repurposing’/exp OR ‘drug rescue’/exp OR ‘off-label use’ OR ‘off-label uses’ OR ‘off-label prescribing’/exp OR ‘unlabeled indication’ OR ‘high throughput screening’/exp OR ‘high throughput screening assays’/exp) AND (“in silico” OR “in silicos” OR “computer simulation” OR “computerized model”))	424
**Web of Science**	TS= ((“drug repositioning” OR “drug repurposing” OR “drug rescue” OR “off-label use” OR “off-label uses” OR “off-label prescribing” OR “unlabeled indication” OR "high throughput screening“ OR "high throughput screening assays") AND (“in silico” OR “in silicos” OR “computer simulation” OR “computerized model”))	220
**Scopus**	TITLE-ABS-KEY ((“drug repositioning” OR “drug repurposing” OR “drug rescue” OR “off-label use” OR “off-label uses” OR “off-label prescribing” OR “unlabeled indication” OR "high throughput screening” OR "high throughput screening assays") AND (“in silico” OR “in silicos” OR “computer simulation” OR “computerized model”)) AND (TITLE-ABS-KEY("2019 nCoV" OR "2019nCoV" OR "2019 novel coronavirus" OR "COVID 19" OR covid19 OR "new coronavirus" OR "novel coronavirus" OR "SARS CoV-2" OR "COVID 19" OR "SARS-CoV" OR "2019-nCoV" OR "SARSCoV-2" OR coronavirus OR covid OR "COVID-19" OR "social distance"))	500
**Open Grey**	((“drug repositioning” OR “drug repurposing” OR “drug rescue” OR “off-label use” OR “off-label uses” OR “off-label prescribing” OR “unlabeled indication” OR "high throughput screening” OR "high throughput screening assays") AND (“in silico” OR “in silicos” OR “computer simulation” OR “computerized model”))	0
