# Supplementary material for: In silico approaches for drug repurposing in oncology: a scoping review
Source: Front Pharmacol. 2024 Jun 11;15:1400029. doi: 10.3389/fphar.2024.1400029 (PMC11196849; doi:10.3389/fphar.2024.1400029)
Supplement: Supplementary file 2 [file DataSheet3.PDF]

Supplementary File 4. In silico methods used for drug repurposing

Method	Software	Reference
Molecular Modelling	Autodock Vina	https://doi.org/10.1002/jcc.21334
	GLIDE	https://doi.org/10.1021/jm0306430
	MOE	https://doi.org/10.1002/bmb.65
	e-LEA3D	https://doi.org/10.1093/nar/gkq322
	OELib library enumeration	OELib library enumeration. http://www.eyesopen.com
	ChemOffice CombiChem	https://doi.org/10.1021/ja1005306
Signature-based	CMAp	https://doi.org/10.1126/science.1132939
	GoPredict	https://doi.org/10.1186/s13040-016-0097-1
	Gene2Drug	https://doi.org/10.1093/bioinformatics/btx800
	NFFinder	https://doi.org/10.1093/nar/gkv445
	L1000CDS	https://doi.org/10.1038/npjsba.2016.15
Network-based	STITCH	https://doi.org/10.1093/nar/gkm795
	DrugNet	https://doi.org10.1016/j.artmed.2014.11.003
	MBiRW	https://doi.org/10.1093/bioinformatics/btw228
	BalestraWeb	https://doi.org/10.1093/bioinformatics/btu599
	DrugGenEx-Net	https://doi.org/10.1186/s12859-016-1065-y

Learning Approach	BenevolentIA	https://doi.org/10.1016/S0140-6736(20)30304-4
	DeepChem	Deep-learning models for Drug Discovery and Quantum Chemistry. https://github.com/deepchem/deepchem
	Atomwise	https://doi.org/10.48550/arXiv.1510.02855
	deepDR	https://doi.org/10.1093/bioinformatics/btz418
	DTINet	https://doi.org/10.1038/s41467-017-00680-8
	DeepDrug	https://doi.org/10.1101/2020.11.09.375626
	DeepPrupose	https://doi.org/10.48550/arXiv.2004.08919
